# Pathogen-Derived Extracellular Vesicle-Associated Molecules That Affect the Host Immune System: An Overview

**DOI:** 10.3389/fmicb.2018.02182

**Published:** 2018-09-12

**Authors:** Marije E. Kuipers, Cornelis H. Hokke, Hermelijn H. Smits, Esther N. M. Nolte-‘t Hoen

**Affiliations:** ^1^Department of Parasitology, Leiden University Medical Center, Leiden, Netherlands; ^2^Department of Biochemistry and Cell Biology, Utrecht University, Utrecht, Netherlands

**Keywords:** extracellular vesicles, pathogen, host, immune response, lipid, protein, nucleic acid, glycan

## Abstract

Recently, the interest in extracellular vesicles (EVs) released by pathogens like bacteria, fungi, and parasites has rapidly increased. Many of these pathogens actively modulate the immune responses of their host and there is accumulating evidence that pathogen-derived EV contribute to this process. The effects of pathogen-derived EV on the host immune system have been attributed to proteins, lipids, nucleic acids, and glycans contained in, or present on these EV. For example, toxins in bacterial EV can modulate pathogen clearance and antigen presentation, while EV-associated polysaccharides are potential vaccine targets because they induce protective immune responses. Furthermore, parasite EV-associated microRNA may increase parasite survival via host gene repression, and the lipid A moiety of LPS in bacteria-derived EV induces strong pro-inflammatory responses. Research on pathogen EV-associated molecules may pave new avenues to combat infectious diseases by immune intervention. This review provides an overview of the current knowledge of EV-associated molecules released by extracellular pathogens and their effects on the host immune system. The current focus and future hotspots of this rapidly expanding field will be highlighted and discussed.

## Introduction

An increasing number of studies documenting the molecular characteristics and the function of pathogen-derived extracellular vesicles (EVs) suggest that pathogen EV play important roles in the activation and modulation of the host immune system. However, only few reports assign EV-mediated effects to specific EV-associated molecules or molecular principles. Here, we focus on defined pathogen-derived EV-associated molecules, their immunomodulatory effects, and the implications for host–pathogen interactions. We first briefly discuss pathogen–host communication via EV and EV biogenesis. Next, various classes of molecules in pathogen-derived EV—proteins, lipids, glycans (carbohydrate chains), and nucleic acids—will be addressed with respect to molecular identity and function. With this approach we aim to outline the current understanding of molecular principles through which pathogen EV modulate immune responses of their hosts.

## Pathogen–Host Communication: Sending Messages Via Extracellular Vesicles

Many pathogens, including bacteria, fungi and parasites, have evolved to survive and reproduce in their host environment. For their success, modulation of the host immune system is often crucial. Simultaneously, the mammalian innate immune system has evolved to recognize and respond to the invading pathogens in order to eliminate them. Various pathogen-associated molecular patterns (PAMPs)—such as proteins, lipids, glycans, and nucleic acids—can bind to different families of pattern recognition receptors (PRRs), including Toll-like receptors (TLRs), C-type lectin receptors (CLRs), and NOD-like receptors (NLRs), expressed by both immune and non-immune cells in the host. While the activation of PRRs by PAMPs is essential for immunity and host defense, pathogens have developed several modulatory mechanisms to interfere in PRR binding and signaling (Takeuchi and Akira, 2010; Kumar et al., 2011; Blander and Sander, 2012). In addition to PAMPs, pathogens can employ other molecules to increase their survival. For instance, cytotoxic proteins that induce apoptosis, enzymes targeting intracellular signaling pathways, or host gene regulating micro(mi)RNAs (Santos and Finlay, 2015; Poole et al., 2018). Classically, PAMPs and other pathogen-derived immunomodulatory molecules are found on the surface of pathogens or they are released as secretory biomolecules. Recently, however, it has become clear that PAMPs and other immunomodulatory molecules can also be released into the extracellular space as part of EV.

Extracellular vesicles are a collective term for exosomes, microvesicles, and other cell-derived membrane-enclosed vesicles. These vesicles transport various molecules, including proteins, lipids, and nucleic acids between cells within one organism or between organisms, such as in host–pathogen cross-talk. Mammalian EV contain a mix of endosomal and plasma membrane proteins, including tetraspanins and major histocompatibility complex (MHC) molecules, and cytosolic proteins such as cytoskeletal proteins and heat shock proteins (Yanez-Mo et al., 2015). Lipids are essential for the formation and structure of the EV bilayer and EV are mainly enriched in cholesterol, sphingomyelin, phosphatidylserine, and glycosphingolipids. In addition, mammalian EV can transfer bioactive lipids and lipid-related enzymes between cells, such as prostaglandin E2 and phospholipase A2, respectively (Record et al., 2014). The most abundant nucleic acids in EV are small RNA species (<200 nucleotides) (Nolte-‘t Hoen et al., 2012), but mRNA, long non-coding RNA, and DNA have also been detected (Yanez-Mo et al., 2015). A lot of research has been dedicated to the characterization of EV-derived miRNA because of their well-known capacity to modulate gene expression. Additionally, EV contain glycans (Gerlach and Griffin, 2016) that are present as part of larger glycoconjugates, such as glycolipids and proteoglycans, or as post-translational modification of proteins. Given that the variation and heterogeneity in glycoconjugates is very high, EV-associated glycans may serve multiple and dedicated roles in both the biogenesis and function of EV (Costa, 2017). The molecular composition of mammalian- and pathogen-derived EV is highly dynamic and depends on the type of EV-producing cell/organism, the developmental/physiological status of that cell/organism, and environmental conditions (Yanez-Mo et al., 2015). Therefore, the functional behavior of these EV and their effects on recipient cells are probably ‘donor’ cell/organism- and niche/context-specific (Nolte-‘t Hoen and Wauben, 2012). The immunological imprinting by EV has been spearheaded by previous studies on EV derived from mammalian immune cells (reviewed in Thery et al., 2009; Nolte-‘t Hoen and Wauben, 2012; Robbins and Morelli, 2014), tumor cells (reviewed in Tkach and Thery, 2016; Wendler et al., 2016) and mesenchymal stromal/stem cells (reviewed in Bruno et al., 2015; Lener et al., 2015; Stephen et al., 2016).

The release of pathogen-derived vesicles was observed 40–50 years ago in gram-negative bacteria, *Vibrio cholerae* (Chatterjee and Das, 1967) and *Neisseria meningitidis* (Devoe and Gilchrist, 1973), the fungus *Cryptococcus neoformans* (Takeo et al., 1973), and the parasites *Schistosoma mansoni* and *Fasciola hepatica* (Senft et al., 1961; Threadgold, 1963). These vesicles were initially regarded as artifacts, until decades later, studies on gram-negative bacteria showed that their released outer membrane vesicles supported bacterial survival. Indeed, bacterial vesicles contributed to biofilm formation (Schooling and Beveridge, 2006) and were able to transfer DNA to other bacteria, thereby sharing genes involved in, for example, antibiotic resistance (Renelli et al., 2004; Mashburn-Warren and Whiteley, 2006). In the past decade, evidence was also provided for release of EV by gram-positive bacteria (Dorward and Garon, 1990), fungi (Rodrigues et al., 2007), parasites (Silverman et al., 2010), parasite-infected cells, like *Plasmodium falciparum*-infected red blood cells (Mantel et al., 2013), and the pathogenic protozoa *Acanthamoeba castellanii* (Goncalves et al., 2018). The description of EV released by pathogen-infected cells is beyond the scope of this review and has been extensively described elsewhere (Schwab et al., 2015; Nolte-‘t Hoen et al., 2016; Rodriguez and Prados-Rosales, 2016; Szempruch et al., 2016). In this review, we focus on EV released by extracellular pathogens and their direct effects on the host.

## Extracellular Vesicle Biogenesis in Mammals and Pathogens

**FIGURE 1:**
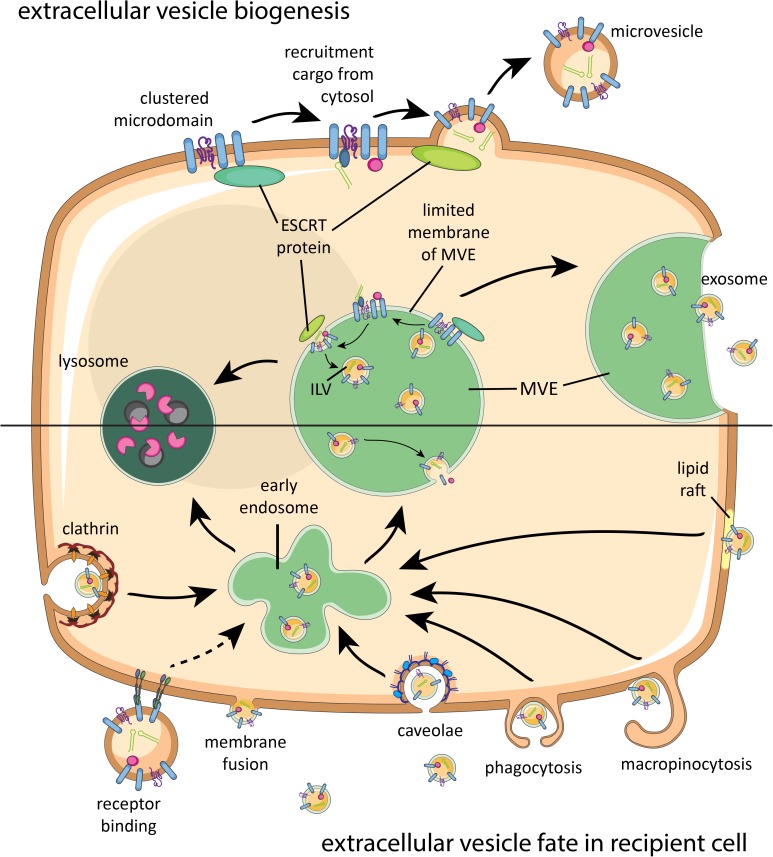
Brief schematic summary of known mammalian extracellular vesicle (EV) biogenesis and their fate in recipient cells. Proteins, lipids, and sorting machineries cluster in microdomains of the plasma membrane or limited membrane of the multivesicular endosome (MVE) and can recruit molecules from the cytosol **(top)**. Furthermore, components of the sorting machinery contribute to microvesicle or intraluminal vesicle (ILV) formation. Microvesicles bud off the plasma membrane, while ILVs are released as exosomes once the MVE fuses with the plasma membrane. Released EV can interact with a recipient cell via receptor binding, fusion, or various internalization routes **(bottom)**. After endocytosis, EV will be either degraded in lysosomes or end up in MVEs with endogenous ILVs. Intraluminal cargo can only be released into the cytosol of the recipient cell upon fusion of EV with the plasma membrane or MVE membrane. ESCRT, endosomal sorting complex required for transport.

Despite growing numbers of studies on EV biogenesis in mammalian cells, full details of the molecular pathways involved are not yet resolved (van Niel et al., 2018). So far, it is known that various sorting machineries are involved in clustering membrane-associated proteins and lipids destined for secretion via EV. This clustering occurs within membrane microdomains found on the limited membrane of the multivesicular endosome (MVE, also called multivesicular body) and on the plasma membrane (**Figure 1**). Although the biogenesis of vesicles within the MVE is better understood than vesicle biogenesis at the plasma membrane, both routes share molecular machineries for EV generation, such as ‘Endosomal Sorting Complex Required for Transport’ (ESCRT) proteins and tetraspanins. Both ESCRT-dependent and independent mechanisms have been implicated in clustering of cargo for biogenesis of EV (Colombo et al., 2014). The microdomain-associated molecules have also been suggested to participate in recruiting soluble proteins and ribonucleoprotein complexes from the cytosol into the vesicles. Budding of EV from the plasma membrane or into the lumen of MVE is often ESCRT dependent. The type of recruited sorting machinery likely determines whether the MVE will fuse with the plasma membrane or with lysosomes (Ostrowski et al., 2010). Upon fusion of the MVE with the plasma membrane, the intraluminal vesicles (ILVs) are released into the extracellular space and then referred to as exosomes. EV budding from the plasma membrane are often called microvesicles. A schematic overview of EV biogenesis pathways is shown in **Figure 1 (top)**. For further details on the biogenesis of mammalian EV, we refer to a recently published comprehensive review on this topic (van Niel et al., 2018).

With regard to pathogen-derived EV, the release of EV by parasites shows most similarities with EV release by mammalian cells. Transmission electron microscopic (TEM) analysis of multicellular parasites, such as *F. hepatica* (de la Torre-Escudero et al., 2016), and unicellular protozoan parasites, such as *Trypanosoma cruzi* (Bayer-Santos et al., 2013), indicated budding of microvesicles from the plasma membrane and the presence of ILVs in MVEs. Interestingly, a third secretion route was described for *T. cruzi* and *Leishmania* spp. where vesicles are released from an organelle involved in protein endocytosis and exocytosis called the flagellar pocket (Silverman et al., 2008; Bayer-Santos et al., 2013; Atayde et al., 2015). However, whether these flagellar membrane-derived EV differ in molecular content compared to the EV released via the other two routes remains unknown (Szempruch et al., 2016). In addition, proteomic studies on EV from many different parasite species (excluding the strictly intracellular parasites) have indicated the presence of molecular orthologs of proteins involved in EV biogenesis, including ESCRT components, the tetraspanin CD63, and vacuolar protein sorting-associated protein 4 (VPS4) (Mantel and Marti, 2014; de la Torre-Escudero et al., 2016). These findings suggest that EV formation is highly conserved in eukaryotes. It is less clear how fungi-derived vesicles are formed. TEM of *C. neoformans* showed MVE-like structures and ESCRT proteins have been detected in EV from *Saccharomyces cerevisiae*. ESCRT proteins were not required for EV release in *S. cerevisiae*, but played a role in determining the protein composition of EV (Rodrigues et al., 2015). Furthermore, it is not known how fungal EV traverse the complex fungal cell wall. For fungi and other thick-walled microorganisms, such as mycobacteria and gram-positive bacteria, it has been suggested that EV traverse the wall by (1) mechanical pressure that forces EV through small pores in the cell wall; (2) passage through protein channels in the cell wall; and (3) remodeling/degradation of the wall by EV-associated enzymes (detected in proteomic analyses) (Brown et al., 2015). Different models have also been proposed to explain the biogenesis of gram-negative bacteria-derived outer membrane vesicles (reviewed in Pathirana and Kaparakis-Liaskos, 2016; Jain and Pillai, 2017). For this class of bacteria, it remains to be elucidated whether outer membrane vesicles originate from the inner and/or outer membrane, or both (Jain and Pillai, 2017). Since the subcellular origin of vesicles released by pathogens is generally unknown, we will here refer to all types of pathogen-derived vesicles as EV.

## Ev-Associated Molecules and Targeting to Recipient Cells

There is limited knowledge on the mechanisms through which EV can interact with cell surfaces and transfer their (intraluminal) cargo to the recipient cell. In general, membrane-bound (glyco)proteins and (glyco)lipids drive the initial interaction with the recipient cell. First, the EV needs to dock at the recipient plasma membrane where it can subsequently remain bound to the surface, be internalized, or fuse with the cell membrane. Receptors on the target cell plasma membrane, such as integrins, can recognize the exterior cargo of the EV, which can lead to activation of signaling cascades within the cell (van Niel et al., 2018). Receptor binding may also facilitate endocytosis of the EV, a route that targets the EV to endosomes and often further to degradation in lysosomes. In addition to receptor-mediated internalization, mammalian EV can also be endocytosed via non-specific macropinocytosis, clathrin-mediated endocytosis, and endocytosis via lipid rafts or caveolae (van Niel et al., 2018). EV need to fuse with the limited membrane of the endosome or with the plasma membrane in order to deliver their intraluminal cargo such as nucleic acids, eicosanoids, and soluble proteins to the cytosol of recipient cells. A simplified overview of EV interaction with recipient cells can be found in **Figure 1 (bottom)**.

Pathogen-derived EV can interact with host cells in various ways. For example, EV from gram-negative bacteria were shown to either fuse with the membrane of the recipient cells or internalize via all previous mentioned routes of endocytosis (O’Donoghue and Krachler, 2016). Additionally, defined molecules on the surface of pathogen-derived EV may drive their targeting to specific cells or organs in the host to create a niche for infection and survival. Since PAMPs are abundantly present on the surface of pathogens (Broz and Monack, 2013), EV that bud off these membranes likely interact with PRRs on recipient cells, as was demonstrated for bacteria-derived EV (Pathirana and Kaparakis-Liaskos, 2016; Hashimoto et al., 2017; Canas et al., 2018). The presence of pathogen-specific enzymes, toxins, or PAMP-containing molecules distinguishes pathogen-derived EV from those released by mammalian cells and may explain differences in their uptake and function. Pathogen-derived molecules in or on EV may therefore be exploited in biomarker research and therapeutic applications. Examples include the detection of parasite-derived EV-associated miRNA in host serum as diagnostic marker for schistosome infections and the exploration of new drugs that target the fusion of *Trypanosome* EV to erythrocytes for prevention of anemia (Zhang et al., 2018).

## Effects of Pathogen Ev-Associated Molecules on the Immune System

Below, we give an overview of immunomodulatory effects induced by components of pathogen-derived EV, subdivided per major class of biological macromolecules (proteins, lipids, glycans, and nucleic acids). In this way, we aim to assess whether there are similarities in how different pathogens use EV-associated molecules to affect host cells. This overview is restricted to studies in which clear evidence was provided for the association of a pathogen-specific molecule in/on EV and the direct involvement of this molecule in the EV-induced effects. A total overview of the selected papers, the reported EV-associated molecules, the observed effects on the host immune system, and the methodological approaches applied in these studies can be found in **Table 1**.

**Table 1:** Pathogen EV-associated molecules and their effect on host immunity.

Microbe/genus	Species (strain)	EV-associated molecule	Effect on host	Methodological approach ^a^	Reference
**Bacteria**					
**gram-negative**					
*Escherichia*	*coli* (CP9)	CNF1 toxin	Attenuates antimicrobial function and chemotaxis of murine neutrophils	Density gradient, proteinase K, mutant strain	Davis et al., 2006
	*coli* (LB226692)	Shiga toxin 2a	Activates caspase- 9 and caspase-3 in human intestinal epithelial cells resulting in apoptosis	Density gradient, proteinase K, electron microscopy, mutant strain, blocking control internalization	Kunsmann et al., 2015
		H4 flagellin	Induces IL-8 secretion by human intestinal epithelial cells		
	*coli* (BL21)	LPS (lipid A)	EV target LPS to cytosol and induce IL1-β release via caspase-11 activation in murine cells *in vitro* and *in vivo*	Electron microscopy^a^, mutant strain^a,b^, blocking control^a^, liposomes^b^, internalization^a,b^	^a^Vanaja et al., 2016; ^b^Santos et al., 2018
	*coli* (JC8031)	Less acetylated PNAG polysaccharide	Immunized mice show protective immune responses after challenge with unrelated bacteria	Electron microscopy, mutant strain	Stevenson et al., 2018
*Francisella*	*t. holarctica* (LVS)	FtlA lipase protein	Facilitates adhesion to and internalization of bacteria by murine macrophages	Density gradient, proteinase K, mutant strain	Chen et al., 2017
*Helicobacter*	*pylori* (251)	Peptidoglycan (PGN)	Promotes inflammation *in vitro* and *in vivo* via NOD1-dependent mechanism	Density gradient, proteinase K, DNase, mutant strain	Kaparakis et al., 2010
*Moraxella*	*catarrhalis* (BBH18)	*Moraxella* IgD-binding protein	B cell receptor binding and vesicle internalization via B cell receptor	Electron microscopy, DNase, mutant strain, blocking control	Vidakovics et al., 2010
		DNA	Increases IL-6 and IgM secretion via TLR9 activation in human tonsillar B cells		
		Ubiquitous surface proteins A1/A2	Downregulation of pro-inflammatory response in epithelial cells	Mutant strain	Schaar et al., 2011
*Neisseria*	*gonorrhoeae* (MS11-A)	PorB porin protein	Targets mitochondria to activate the caspase pathway and apoptosis in murine macrophages	Density gradient, electron microscopy, mutant strain, internalization	Deo et al., 2018
	*meningitidis* (H44/76)	LPS (lipid A)	Activation of innate immunity *in vitro*	Mutant strain	Zariri et al., 2016
*Pseudomonas*	*aeruginosa* (PA14)	Cif toxin	Reduces deubiquitination of CFTR and TAP in airway epithelial cells leading to reduction of pathogen clearance and antigen presentation, respectively	Density gradient, mutant strain, internalization	Bomberger et al., 2011, 2014
		tRNA fragment	Reduces LPS- and EV-induced IL-8 release by human airway epithelial cells. Reduces neutrophil infiltration in mouse lung.	Density gradient, RNase, mutant strain, molecule control, internalization	Koeppen et al., 2016
**gram-positive**					
*Bacillus*	*anthracis* (34F2)	Anthrolysin toxin polypeptide	Contribute to cytotoxicity in murine macrophages and induces IgM responses *in vivo*	Electron microscopy, mutant strain, blocking control, molecule control	Rivera et al., 2010
*Streptococcus*	*pneumoniae*	PspA surface protein	PspA specific IgG responses and increased survival in EV immunized mice	Density gradient, proteinase K, mutant strain, molecule control	Muralinath et al., 2011
**Fungi**					
*Paracoccidioides*	*brasiliensis* (Pb18, Pb3) *lutzii* (Pb01)	Surface glycan	Binds to DC-SIGN in microarray; functional effect not shown	Lipophilic dye, blocking control	Peres da Silva et al., 2015a
**Parasites**					
**helminths**					
*Heligmosomoides*	*polygyrus*	miRNA	Repression of *Dusp1* to increase parasite survival in host	m RNase, lipophilic dye, molecule control, internalization	Buck et al., 2014
*Opisthorchis*	*viverrini*	Tetraspanin (TSP-1) protein	Involved in EV uptake and subsequent induction of IL-6 production by human cholangiocytes	Blocking control	Chaiyadet et al., 2015
**protozoa**					
*Leishmania*	*Major, donovani*	GP63 metalloprotease	Role in *Leishmania* EV protein sorting which indirectly dampens immune responses. Reduction of miR-122 expression in mouse liver cells to favor infection.	Density gradient^d^, mutant strain^d^, blocking control^c^, liposomes^c^, molecule control^c^	^c^Ghosh et al., 2013; ^d^Hassani et al., 2014
*Trypanosoma*	*cruzi*	tRNA- halves	Increases expression of *il-6* and *cxcl2* in HeLa cells	Molecule control, internalization	Garcia-Silva et al., 2014


*^1^Brief description of methodological approaches: **density gradient**, density gradient isolation for purification of EV, often combined with Western Blot detection of the EV-associated molecule; **proteinase K, DNase, RNase**, proteinase K, DNase, or RNase treatment of EV, often combined with a detergent or enzyme inhibitor to investigate the topology of the EV-associated molecule; **electron microscopy** electron microscopy immunogold staining to show association of the molecule with EV; **lipophilic dye**, staining of EV membrane or EV-depleted control samples with lipophilic dye for binding/uptake assays; **mutant strain**, EV from mutant strains lacking the (functional) molecule; **blocking control**, using a control to inhibit the EV-associated molecule with either a neutralizing antibody or compound; **liposomes**, mimicking EV by synthesis of liposomes containing only the molecule of interest; **molecule control**, using (a synthetic form of) the molecule as a soluble protein control to confirm the importance of EV association; **internalization**, confirmed intracellular detection of the molecule in recipient cells incubated with the EV. a–d, methodological approaches applied by the indicated references.*

### Pathogen EV-Associated Proteins With Immunomodulatory Activities

**FIGURE 2:**
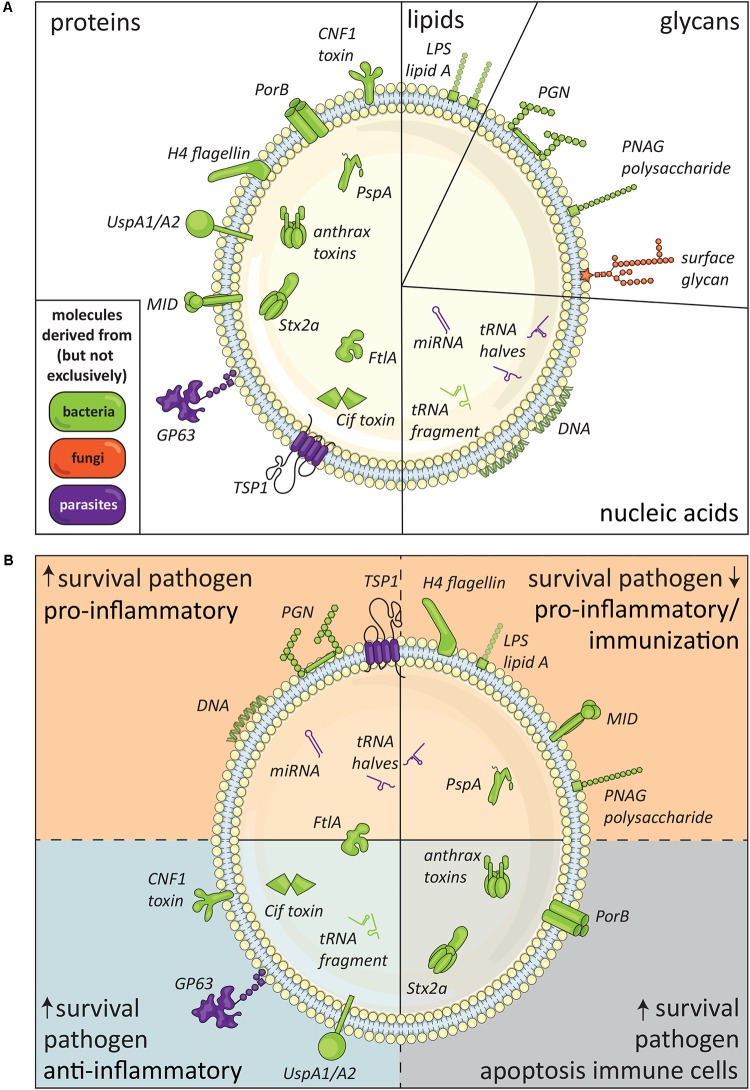
Immunomodulatory molecules in pathogen-derived EV. **(A)** Schematic summary of pathogen-derived EV-associated molecules with immunomodulatory effects released by bacteria (green), fungi (orange), and parasites (purple), subdivided in proteins, lipids, glycans, and nucleic acids. The suggested topology of the molecules, i.e., inside the EV or in its lipid membrane facing the exterior, is also shown. **(B)** Defined EV-associated molecules released by pathogens arranged based on immunomodulatory effects, i.e., pathogen survival, inflammatory response, and immune cell apoptosis.

Proteins form the largest group of known pathogen EV-associated molecules (**Figure 2A**). These EV proteins have been shown to affect immune responses or the survival rates of the host or pathogen (**Figure 2B**). Below, we distinguish and describe pathogen EV-associated proteins that cause cytotoxicity, increase pathogen survival, or induce pro-inflammatory responses.

#### Cytotoxic Proteins Associated to Pathogen EV

Thus far, only EV-associated proteins derived from bacteria have shown to compromise immune defenses by inducing apoptosis of host cells. For example, EV from the gram-negative bacterium *N. gonorrhoeae* contained a porin protein, PorB, that was shuttled to mitochondria in host macrophages (Deo et al., 2018). Here, the voltage gated pore induced loss of mitochondria membrane potential and cytochrome c release leading to activation of the caspase pathway and apoptosis. As such, PorB promoted immune evasion and survival of this sexually transmitted infection. Importantly, it was shown that *N. gonorrhoeae* depends on EV for the delivery of PorB, suggesting a role for bacteria-derived EV and associated proteins in increased virulence. In addition, various cytotoxic proteins were found in EV derived from a highly virulent *Escherichia coli* strain (Kunsmann et al., 2015). The authors demonstrated the association of Shiga toxin 2a (Stx2a) and Shigella enterotoxin 1 with the EV. Stx2a was shown to cause cytotoxicity after EV uptake by various human intestinal epithelial cell lines via activation of caspase-9 and caspase-3. This effect could not be reduced by preincubating the EV with a Stx2a-neutralizing antibody, confirming the intravesicular localization of Stx2a (Kunsmann et al., 2015).

Extracellular vesicle released by the gram-positive bacteria *Bacillus anthracis* were also shown to contain cytotoxic molecules (Rivera et al., 2010). The anthrax toxins released by this bacterium have lethal consequences for its host during infection. Both EV-associated anthrolysin (a cholesterol-dependent pore-forming protein) and an anthrax toxin polypeptide induced toxicity in macrophages. Additional effects of these EV-associated toxins were shown in immunized mice. Immunization with isolated EV from *B. anthracis* increased the survival of mice after bacterial re-challenge and led to an increase in IgM titers against *B. anthracis* toxins in mouse sera. This differed from immunization with purified, non-EV associated toxins, which mainly induced IgG production (Rivera et al., 2010).

#### EV-Associated Proteins Promoting Survival of Pathogens

Bacteria-derived toxins transported by EV can also modulate immune cell functions to promote pathogen survival. For example, cytotoxic necrotizing factor type 1 (CNF1) in EV from an uropathogenic *E. coli* was shown to reduce antimicrobial activity and chemotactic responses in neutrophils (Davis et al., 2006). Another example has been provided for the Cif toxin derived from the gram-negative bacterium *Pseudomonas aeruginosa* (Bomberger et al., 2011, 2014). EV-associated Cif increased the bacterial pathogenesis of *P. aeruginosa* by reducing the abundance of the cystic fibrosis transmembrane conductance regulator (CFTR) in human airway epithelial cells. CFTR facilitates chloride secretion by epithelial cells needed for mucociliary clearance of the pathogen in the lungs (Bomberger et al., 2011). In a follow-up paper, it was suggested that Cif may also increase the severity and duration of infections in the airways by reducing the ‘transporter associated with antigen processing 1’ (TAP1). This leads to reduced antigen transport to MHC class I molecules and inhibition of antigen presentation, including antigens from other pathogens such as viruses (Bomberger et al., 2014). These studies suggest that Cif can contribute to pathogen survival in various ways. Another human respiratory pathogen, *Moraxella catarrhalis*, was shown to release EV that reduced TLR2-induced IL-8 production by human alveolar epithelial cells, via the activity of EV-associated Ubiquitous surface protein (Usp)A1. UspA1 can bind to carcinoembryonic antigen-related cell adhesion molecule-1 (CEACAM-1) on epithelial cells and subsequently reduce TLR2-activated IL-8 production (Schaar et al., 2011). As IL-8 is a key player to attract other cells, mainly neutrophils, to the site of infection, UspA1-dependent reduction of IL-8 could increase bacterial survival in the lungs.

Extracellular vesicle from zoonotic *Francisella tularensis*, the gram-negative bacterium that can cause fetal pneumonic tularemia in humans, contain a protein with lipolytic activity named FtlA. Since this bacterium primarily replicates inside alveolar macrophages and epithelial cells, the success of invasion into host cells determines its virulence. *FtlA* mutant bacteria showed significantly reduced lipolytic activity and virulence and reduced inflammatory cell infiltrations in a lung infection model (Chen et al., 2017). Interestingly, it was shown that FtlA is absent from the surface of the bacterium but present on the surface of EV from *F. tularensis*. By adding EV from the wild type strain to *ftlA* mutant bacteria *in vitro*, bacterial infiltration increased significantly, suggesting a role for the EV-associated lipase protein in bacterial adhesion and cell invasion (Chen et al., 2017).

In addition to gram-negative bacteria, parasites also release EV that promote survival in the host. This was shown for EV containing Glycoprotein (GP)63 released by the protozoan parasite *L. major* (Hassani et al., 2014). GP63 is a zinc-dependent metalloprotease that is expressed on the surface of the infectious *Leishmania* life stage. Parasite-derived GP63 is known to modulate signaling pathways (such as tyrosine phosphatases) within macrophages such that it inhibits inflammatory responses leading to increased parasite survival. Although this is an intracellular parasite, the authors used isolated EV from *in vitro* extracellular parasite cultures. *In vivo* administration of EV from GP63^-/-^ *L. major* parasites resulted in stronger pro-inflammatory effects, such as increased neutrophil and eosinophil recruitment, compared to wild type *L. major*. This suggests that EV-associated GP63 could dampen immune responses and may support the establishment of *Leishmania* infection in the host. However, these effects could also be due to other EV-associated proteins, since it was shown that GP63 additionally affects the proteomic content of EV (Hassani et al., 2014). Furthermore, EV-associated GP63 was shown to affect liver cell function. GP63-containing EV released by *L. donovani* reduced the activity of miRNA miR-122 in a human liver cell line, likely by affecting DICER1-mediated cleavage of this miRNA (Ghosh et al., 2013). These results were confirmed *in vivo* by administering liposomes containing purified GP63 or *Leishmania*-derived EV to mice. The GP63-induced reduction in DICER1 and miR-122 expression in hepatocytes resulted in metabolic changes in these cells and lower serum cholesterol levels. This may favor parasite infection, as a high serum cholesterol is associated with reduced parasite burden in the liver (Ghosh et al., 2013).

#### Pro-inflammatory Responses Induced by EV-Associated Proteins

In addition to their cytotoxic and survival-promoting effects, pathogen-derived EV have also been implicated in raising host immune responses against the pathogens. EV from the liver fluke (flatworm) *Opisthorchis viverrini*, for example, were shown to induce IL-6 production by human cholangiocytes, an effect that could be blocked by antibodies against the EV-associated tetraspanin TSP-1 (Chaiyadet et al., 2015). This suggested a role for TSP-1 carrying EV in the chronic inflammatory environment observed in host tissues during infection with *O. viverrini*. Also EV-associated proteins from bacteria can induce pro-inflammatory responses in the host. This has been shown for virulent *E. coli*-derived EV-associated H4 flagellin, which triggered TLR5 signaling and subsequent IL-8 release by EV-targeted intestinal cells (Kunsmann et al., 2015). An additional way in which pro-inflammatory responses are induced by EV was shown for *M. catarrhalis*. *Moraxella* IgD-binding (MID) protein on the EV of this gram-negative bacterium was essential for EV uptake by human tonsillar B lymphocytes via the B cell receptor and induced IL-6 production by these cells (Vidakovics et al., 2010). Interestingly, IgM production was increased after B cell uptake of EV, but these antibodies were not specific for *Moraxella* bacteria. The authors therefore suggested that the non-specific IgM production induced by MID-associated EV is rather a survival strategy to divert the B cell antibody production away from the bacterial infection (Vidakovics et al., 2010). These results suggest that one EV-associated protein may induce multiple effects that could benefit both the host and the pathogen.

Overall, these findings indicate that a wide variety of pathogen-derived EV-associated proteins can modulate host immune responses. The pro-inflammatory properties of pathogen-derived EV may be used for EV-based vaccination strategies. This has been investigated for PspA, a surface protein expressed by all strains of the gram-positive *Streptococcus pneumoniae*. PspA was introduced into a gram-negative *Salmonella enterica* strain (Muralinath et al., 2011), after which EV released by these bacteria were used for intranasal immunization of mice. These EV induced a PspA-specific IgG responses and increased mouse survival after *S. pneumoniae* challenge. This was not observed when administrating EV from a similar *Salmonella* strain without PspA or when PspA was administered as purified antigen (Muralinath et al., 2011).

### EV-Associated Lipids

Bacterial LPS is the most intensely investigated pathogen-derived (glyco)lipid associated with EV. LPS is known to bind to TLR4, but can also be recognized in the cytosol by the murine receptor protease caspase-11, and in humans by caspase-4 and caspase-5. EV from gram-negative bacteria were shown to deliver LPS to the cytosol, thereby inducing an inflammatory form of programmed cell death (pyroptosis) and increased IL-1β expression in a caspase-11-dependent manner (Vanaja et al., 2016). These effects were reduced when LPS on EV was neutralized, when EV from an *E. coli* mutant lacking a functional lipid A (an essential component of LPS) were used, or when cells were incubated with EV from gram-positive bacteria lacking LPS. The pro-inflammatory effects were also reduced when using bacteria from a *E. coli* strain which released less EV due to mutations in EV biogenesis. These data suggest a role for EV in delivering LPS into the cytosol and subsequent activation of innate immune responses during an infection with gram-negative bacteria (Vanaja et al., 2016). A more recent study confirmed these findings by mimicking the EV structure of gram-negative bacteria by incorporation of LPS in liposomes (Santos et al., 2018). When stimulating murine macrophages with these liposomes, cell death and IL-1β release were induced in a caspase-11-dependent fashion. In addition, a role for guanylate-binding proteins (GBPs) in this process was observed. GBPs can destroy the membrane of pathogens in the cytosol. It was suggested that EV-associated LPS recruits GBPs, which then engage in a membrane-disrupting activity leading to either LPS release from the EV or providing direct access of caspase-11 to the lipid A moiety of LPS, both resulting in caspase-11 activation (Santos et al., 2018). Lipid A was indeed shown to be essential for GBP-dependent cell death. Additionally, injection of LPS-containing *E. coli*-derived EV into wild type mice caused lethality, while GBP^-/-^ mice showed increased survival and caspase-11 deficient mice showed complete survival. This suggests a role for EV-associated LPS in EV-induced endotoxic shock.

Extracellular vesicle-associated lipids are also gaining attention in the development of EV-based vaccines (Lener et al., 2015). EV derived from the gram-negative bacterium *N. meningitidis* are already used in a vaccine against meningococcal disease in children (Acevedo et al., 2014). Research has been performed on how different types and modifications of LPS in EV from gram-negative bacteria contribute to immunogenicity or (endo)toxicity (Vanaja et al., 2016). Lipid A modifications in *N. meningitidis* mutants, for example, were shown to reduce EV-induced innate immune responses *in vitro*, which could indicate these EV are less endotoxic and thus probably safer to use in vaccines (Zariri et al., 2016).

Thus far, only the above described studies gave strong proof of the presence of biologically active (glyco)lipids in pathogen-derived EV (see **Figure 2A**). Considering the increasing interest in EV and continuous improvement of EV isolation and characterization technologies, it is suspected that additional biological effects of pathogen-derived EV-associated lipids may be elucidated in the near future. For example, mass spectrometry indicated that EV from the parasitic worm *Heligmosomoides polygyrus* differed in lipid composition from murine cell-derived EV and were enriched in ether glycerophospholipids called plasmalogens. The authors created artificial vesicles and observed that increasing the plasmalogen content of the vesicles increased the efficiency with which they fused with cells (Simbari et al., 2016). However, it remains to be elucidated whether plasmalogens influence the immunomodulatory property of these EV.

### Glycans Associated to EV

Several lines of evidence indicate that glycans are present on EV from bacteria, parasites, and fungi. Pathogen glycans can bind to and signal via different host cell receptors, which may lead to modification of host cell function. For example, using mammalian lectin microarrays, it was shown that surface glycans on EV of the fungi *Paracoccidioides brasiliensis* and *P. lutzii* were recognized by human DC-SIGN (Peres da Silva et al., 2015a). In addition, analysis of the proteomes of parasite-derived EV has indicated the presence of many (putative) glycoproteins (Twu et al., 2013; Nowacki et al., 2015; Samoil et al., 2018). Whether or how these components truly interact with host cells and modify their function it is not yet known.

While no data are available on potential immunomodulatory effects of glycans associated to fungi- or parasite-derived EV, several studies addressed the function of glycans on bacteria-derived EV. Mice immunized with EV from a genetically engineered *E. coli* with decreased acetylation of poly-*N*-acetyl-D-glucosamine (PNAG) showed higher protective immune responses after bacterial challenge compared to their highly acetylated counterparts (Stevenson et al., 2018). PNAG is a surface polysaccharide present in many different pathogen species, suggesting that these engineered EV as immunization strategy may be broadly effective. Indeed, immunized mice were protected against lethal doses of *Staphylococcus aureus* and *F. tularensis*. In another study, EV from *Helicobacter pylori* were shown to induce NF-κB activity and subsequent IL-8 production in human epithelial cell lines in an NOD1-dependent fashion (Kaparakis et al., 2010). Cytosolic NOD1 is known to specifically recognize peptidoglycan, a component of the bacterial cell wall. The authors indeed showed that EV-associated peptidoglycan from gram-negative bacteria could activate NOD1. Interestingly, feeding of mice with EV from *H. pylori* increased gastric gene expression of the chemokine *Cxcl2*, which was shown to be dependent on NOD1 and independent of TLR activation (Kaparakis et al., 2010). However, it remains to be determined whether association of peptidoglycan with EV is required to mediate these effects.

Thus far, only a handful of studies have investigated the glycome of EV, none of which addressed pathogen EV (reviewed by Gerlach and Griffin, 2016; Williams et al., 2018), partly due to the technical limitations dictated by the scale and purity of EV preparations. The importance of analyzing the glycomic profiles of EV has been demonstrated for mammalian EV. In different pathological conditions, such as cancer or metabolic diseases, differences were observed in the EV glycomic profile (Escrevente et al., 2011; Staubach et al., 2012; Gerlach et al., 2013), suggesting the physiological importance of EV glycosylation. Studying glycomic profiles of pathogen-derived EV could improve our understanding of EV biogenesis and interaction with recipient cells, and elucidate whether glycosylation of proteins and lipids plays a role in (immune) modulation by these EV (Williams et al., 2018).

### EV-Associated Nucleic Acids

The nucleic acid content of pathogen-derived EV consists of different RNA classes and DNA. Depending on the type of nucleic acid and its localization in the EV these molecules can either regulate host mRNA or trigger RNA or DNA sensing receptors. The latter was observed for EV of the gram-negative bacterium *M. catarrhalis* (Vidakovics et al., 2010). These vesicles were shown to induce the proliferation of human tonsillar B cells and activate TLR9 via its EV-associated DNA that contained CpG-motifs. Furthermore, it was observed that the increase in B cell proliferation was significantly reduced when EV were treated with DNase, suggesting that the DNA is on the outside surface of *M. catarrhalis*-released EV (Vidakovics et al., 2010).

There is an increasing number of studies that report on the composition of the small RNA repertoires in vesicles from bacteria (Blenkiron et al., 2016; Koeppen et al., 2016), fungi (Peres da Silva et al., 2015b), and parasites (Bernal et al., 2014; Buck et al., 2014; Garcia-Silva et al., 2014; Hansen et al., 2015; Nowacki et al., 2015; Zamanian et al., 2015; Zhu et al., 2016). However, so far, only few studies provided evidence that EV-associated RNA is responsible for the modulation of host cells. For example, uptake of EV from the gastrointestinal parasitic worm *H. polygyrus* by mouse epithelial cells led to decreased expression of the *Dusp1* gene while uptake of EV from mouse intestinal cells had no effect (Buck et al., 2014). This effect is likely caused by the EV-associated miRNAs, since transfection of host cells with synthetic analogs of these parasite miRNAs also showed reduction of *Dusp1* expression. DUSP1 is a regulator of mitogen-activated protein kinase (MAPK) signaling and favors a reduction in IL-6 and an increase in macrophage arginase expression. As IL-6 enhances host susceptibility to *H. polygyrus* and arginase is a mediator of killing this parasite in mice, repression of DUSP1 may be a parasite-driven mechanism to enhance its survival in the host. This is a very interesting example of cross-organism communication in which a parasite EV-associated miRNA functions due to complementarity to the host target gene.

Immunomodulatory effects induced by other EV-associated small non-coding RNAs have been described for both parasites and bacteria. The parasite *T. cruzi* releases EV containing transfer RNA-derived small RNAs (tsRNA), that could be visualized inside HeLa cells after uptake of these EV (Garcia-Silva et al., 2014). This led to several alterations in gene expression levels, including increased mRNA levels for pro-inflammatory *il-6* and *cxcl2*. Part of this effect, such as increased *cxcl2* expression, was also observed when HeLa cells were transfected with a synthetic form of the most abundant EV-associated tsRNAs (Garcia-Silva et al., 2014). However, the mechanism by which tsRNA derived from parasite influences host mRNA transcription remains to be elucidated. EV-associated tRNA-derived fragments (from different isoacceptor tRNAs) were also detected in EV from the opportunistic bacterium *P. aeruginosa* (Koeppen et al., 2016). One specific tsRNA (sRNA52320) present in isolated EV could be detected inside EV-treated primary human bronchial epithelial cells. Both EV-mediated transfer of sRNA52320 and direct transfection of cells with synthetic sRNA52320 led to reduction of LPS-induced IL-8 production. This provided indirect evidence that EV-enclosed tRNAs mediated these effects. Also *in vivo*, *P. aeruginosa*-derived EV significantly reduced the mouse homolog of IL-8 in bronchoalveolar lavage fluid and lowered lung neutrophil infiltration, which was sRNA52320 dependent (Koeppen et al., 2016). However, whether the EV-associated tsRNA contributes to persistence of *P. aeruginosa* infections is yet to be determined.

## Conclusion and Outlook

The observed effects of EV released by pathogens underline that intercellular communication via EV is a conserved mechanism that most likely benefits pathogen survival in co-evolution with its host. Immunomodulatory components of pathogen-derived EV are represented in all molecular subclasses—proteins, lipids, glycans, and nucleic acids (**Figure 2A**)—and can modify host cell function or induce host cell apoptosis. Although the field of research on pathogen-derived EV is still in its infancy, many advanced studies have already been performed on EV released by bacteria. The generation of mutant bacterial strains has provided great opportunities to study the function of specific EV-derived molecules. From these studies, we learned that many, if not all, gram-negative bacteria release LPS-containing EV that are well-capable of inducing pro-inflammatory reactions. Despite gaps in our understanding of the broad group of pathogen-derived EV, the currently available data indicate that pathogen EV-associated molecules can promote survival and spreading of pathogens, but can also facilitate the induction of host-immune responses (**Figure 2B**). The exact role of pathogen-derived EV in host–pathogen interaction likely depends on pathogen life stage, environmental, and/or tissue specific conditions.

The current overview does not show us yet whether EV from different pathogens contain conserved classes of molecules or act via similar principles. This is probably due to the limited number of publications on EV-associated immunomodulatory molecules from pathogens that is available at this early stage. In addition, inter-study comparability of data on both mammalian and pathogen EV is currently hampered by the use of a wide range of different techniques to isolate and characterize EV. Different EV isolation methods, for example, yield EV of different purity and can bias toward isolating certain EV subtypes (Mateescu et al., 2017). This urges the need to adhere to guidelines stated in the “minimal information for studies of EV (MISEV)” (Lotvall et al., 2014), of which an update is currently under development. Increasing the reproducibility of EV research also requires that all experimental details relating to EV isolation and characterization are reported in scientific publications. This is facilitated by EV-TRACK, which is a crowdsourcing knowledgebase to centralize experimental parameters of EV publications (Van Deun et al., 2017). Optimization and standardization of experimental methods will help to unravel effects of specific EV-associated molecules from pathogens and is particularly important for the design of, e.g., EV-based vaccines. Additionally, a more standardized approach will allow the comparison of similar molecules or immune responses induced by different pathogen-derived EV.

A promising method to allow pathogen-overarching comparisons of pathogen-derived EV-associated molecules is the use of bioinformatics (Bryant et al., 2017). The increasing number of publications on molecular characterization of pathogen-derived EV will generate large databases, such as EVpedia (Kim et al., 2013), with a wide range of omics data ready to be investigated *in silico*. Computer-based models can search all available pathogen specific EV-associated molecules for family members with immunomodulatory potential that are conserved between different pathogens, and directly point toward interesting molecules for further functional studies. Increasing the knowledge on pathogen-derived EV-associated molecules and their effects on the host immune system will certainly shed further light on the importance of EV molecules in infection biology.

## Author Contributions

MK wrote the manuscript. EN-‘tH, CH, and HS initiated and edited the manuscript. All authors read and approved the final manuscript.

## Conflict of Interest Statement

The authors declare that the research was conducted in the absence of any commercial or financial relationships that could be construed as a potential conflict of interest.
